# 

**Published:** 1894-01

**Authors:** 


### CONTENTS.

PROCEEDINGS.

World's Columbian Dental College—1893 ................................ 1
The Woman's Dental Association of the United States................ 28

COMMUNICATIONS.

The Early Women in Dentistry ................................ 31
An Act to Regulate the Practice of Dentistry in Arizona.............. 35

SELECTIONS.

How to Avoid Taking Cold.............................................. 38
Dentistry in China.............................................. 42
Use of Cocaine.............................................. 43
Diagnosis of Hereditary Syphilis.............................................. 43
Origin and Meaning of the Term "Biology".............................. 45

EDITORIAL.

An Interesting Case.............................................. 47
An Inquiry.............................................. 47
The Ohio State Dental Society.............................................. 49
Mississippi Valley Dental Society.............................................. 50
Bibliographical.............................................. 51

